# Malnutrition and poor oral health status are major risks among primary school children at Lasbela, Balochistan, Pakistan

**DOI:** 10.1186/s41043-017-0100-6

**Published:** 2017-05-19

**Authors:** Muhammad Ayaz Mustufa, Abdul Karim Jamali, Ifra Sameen, Fateh Muhammad Burfat, Mir Yousaf Baloch, Abdul Hameed Baloch, Ghulam Rasool Baloch, Shazia Kulsoom Lashari, Sobiya Mohiuddin Ayaz, Muhammad Younus Baloch

**Affiliations:** 1 5th Floor, PHRC Specialized Research Centre on Child Health, National Institute of Child Health, 75500 Karachi, Pakistan; 2 Mehran Reproductive Health Organization, Karachi, Pakistan; 3 National Institute of Child Health, 75500 Karachi, Pakistan; 4 Faculty of Social Sciences, University of Karachi, Karachi, Pakistan; 5 Department of Health, Government of Balochistan, Quetta, Pakistan; 6 National Rural Support Program (NRSP), Balochistan, Quetta, Pakistan; 7 Jinnah University for Women, Karachi, Pakistan

**Keywords:** Malnutrition, Body mass index, Primary school children

## Abstract

**Background:**

This survey was focusing on health and oral hygiene status of primary school children at Lasbela district considering the comparatively less developed and socio demographically deprived part of the Country.

**Methods:**

A cross sectional survey was conducted to determine the health status of primary school children in seven tehseels of district Lasbela, Balochistan after applying proportionate sampling technique from March 2015 to July 2015. Field teams visited assigned schools to screen children and collect health related data on predesigned and pre coded proforma.

**Results:**

Out of 200 schools, 196 schools found opened, while 2% of schools (04) remained closed. A total of 6363 students were clinically screened. About 45% of the school children had normal body mass index (BMI) and rest were falling in different categories of malnutrition. More than 19% had ear, nose and throat (ENT) problems and around 19% presented with clinical anemia. Less than 50% of children had scar of BCG vaccination and 4% informed about use of gutka/supari chewing (smokeless tobacco use).

**Conclusion:**

In conclusion, we estimated high prevalence of malnutrition, poor oral health including smokeless tobacco use, and low BCG coverage among primary school children at Lasbela. Current scenario suggests immediate and contextually focused interventions to confine existing public health risks and avoid future burden of disease.

## Background

Childhood is a critical period, which has direct impact on economic growth of the country. Early physical, cognitive and socio economic growth constitute foundations of future development. In consequence, retarded health circumstance in early years can be life-long with serious socio economic effects at individual and community level at large. Malnutrition is defined as a state of imbalance of energy or protein and other nutrients leading to measurable adverse effects on human body and clinical outcome [1, 2]. Malnutrition is a universal problem and more rampant in developing countries including Pakistan. It remains undetected and untreated in respective communities due to multiple factors including high cost, social, cultural, environmental, medical, etc.

Under-nutrition is more common in low middle income countries (LMIC). It is reported for stunting and underweight as predisposing factors to deficiency states and infections in children. On the other hand, over-nutrition may subsequent to obesity and other consequences like diabetes, hypertension and heart disease [3–5]. Around 44% Pakistani children (under 5 years of age) are stunted, wasting found that 15.1 and 31.5% children are underweight [5].

According to WHO, obesity is declared as a “global epidemic”. Overeating, sedentary life style, reduced physical activities and high-calorie expenditures are known risk factors in addition to genetic predisposition. This scenario may lead to high morbidity, mortality and escalated economic health expenditure [6, 7]. The economic behavior/income spectrum depends on the nutritional status of an individual. At individual level nutritional status affects health, survival, physical and cognitive functioning with impact on work capacity and productivity [7, 8]. Poverty, on the other hand, having similar root causes all over the world and almost similar consequences like hunger, crime, social unrest, and food insecurity.

In Asian countries, the whole spectrum of malnutrition exists mainly due to wide socioeconomic disparities. Some of these countries face a paradox of underweight and overweight. Poor intrauterine growth resulting in low birth weight followed by rapid childhood weight gain promoting obesity and associated metabolic complications [7, 9–13]. Same paradox has been reported from Philippines, Thailand, and other Asian countries [8, 14–16]. Population below the calorie based food plus non- food poverty line and minimum level of dietary energy consumption is 12.4 and 30%, respectively [17].

Despite the public health impact of childhood malnutrition and the need to monitor trends, there has been little epidemiologic research in remote/rural parts of developing countries like Pakistan and other chronic pediatric health problems is reported due to unavailability of periodic screening mechanism. In the light of above, following survey was conducted to screen school going children in Lasbela, a costal district of Balochistan with an estimated population of more than 1.2 million.

## Methods

### Inception

A pre-survey meeting was convened at Hub, Balochistan. Piloting of study tools was done to further acquaint the survey team with the tools and standard operating guidelines for data collection in a uniform, validated, and reliable manner.

### Access to the community

To enhance compliance and avoid intolerable situations, political / religious community representatives identified in each union council/tehseels as gate keepers. The gate keeper of each representative community was contacted before the start of field activity and informed about the purpose and rationale of the study.

### Enumeration

NRSP, Balochistan provided list of primary schools in consultation with Education Department, Lasbela, Balochistan. Each school visited was enumerated with specific code (NRSP-SP-school list no./date of visit) by a senior field member, under the supervision of a team leader.

### Field visits

Field visits initiated from first week of March 2015 and completed before end of May 2015. Prior to every field visit, gate keeper and community mobilizer of respective community informed about the date and timings of the visit for their presence and facilitation. On average, two to three visits were done by field teams in different intervals in between 8:00 AM and 1:00 PM in each school for maximum coverage.

### Field team

Our field team specifically trained to adopt friendly and pleasing attitude with potential study participants for easy access and confident building behaviors for participation and follow-ups, if required. Keeping in mind that Lasbela district of Balochistan province represents diverse ethnic groups; field team composition was reviewed and rationalized as per need. In addition to the required technical expertise, the field team members intentionally recruited from respective communities to curtail lingual and ethnic barriers and biases.

### Ethical considerations

Respecting to the autonomy of an individual, those who did not agree to participate after introduction and counseling; were excluded as per their desire. Privacy of interviewee was also maintained.

### Monitoring

Following the principle of supportive monitoring, Principal Investigator and Co-Principal Investigator made series of visits to assure quality of data. Furthermore, to avoid conflict of interest at an individual or communal level, ongoing internal monitoring of filled data and verification of physical locations was also done.

### Execution plan

This study was carried out in 200 public primary Schools in Lasbella district, Baluchistan selected through proportionate method. The primary school children irrespective of their age and gender were included. The project team members (data collectors) were trained on project instruments and tools using standardized project manual. All participants received standardized training to fill questionnaires and collect validated and uniform data. A pre tested questionnaire was used and children’s height, weight, ENT, eye assessment, and personal hygiene were recorded after clinical assessment by field doctor. BMI scores were calculated for each children using WHO chart.

Collected data was entered and analyzed in SPSS-20 statistical software. Descriptive statistics of frequency and proportion was used to give a clear picture of baseline characteristics. Along with data of physical parameters including ear, nose, throat, height, weight, and hygiene was screened and worked out.

## Results

**Fig. 1:**
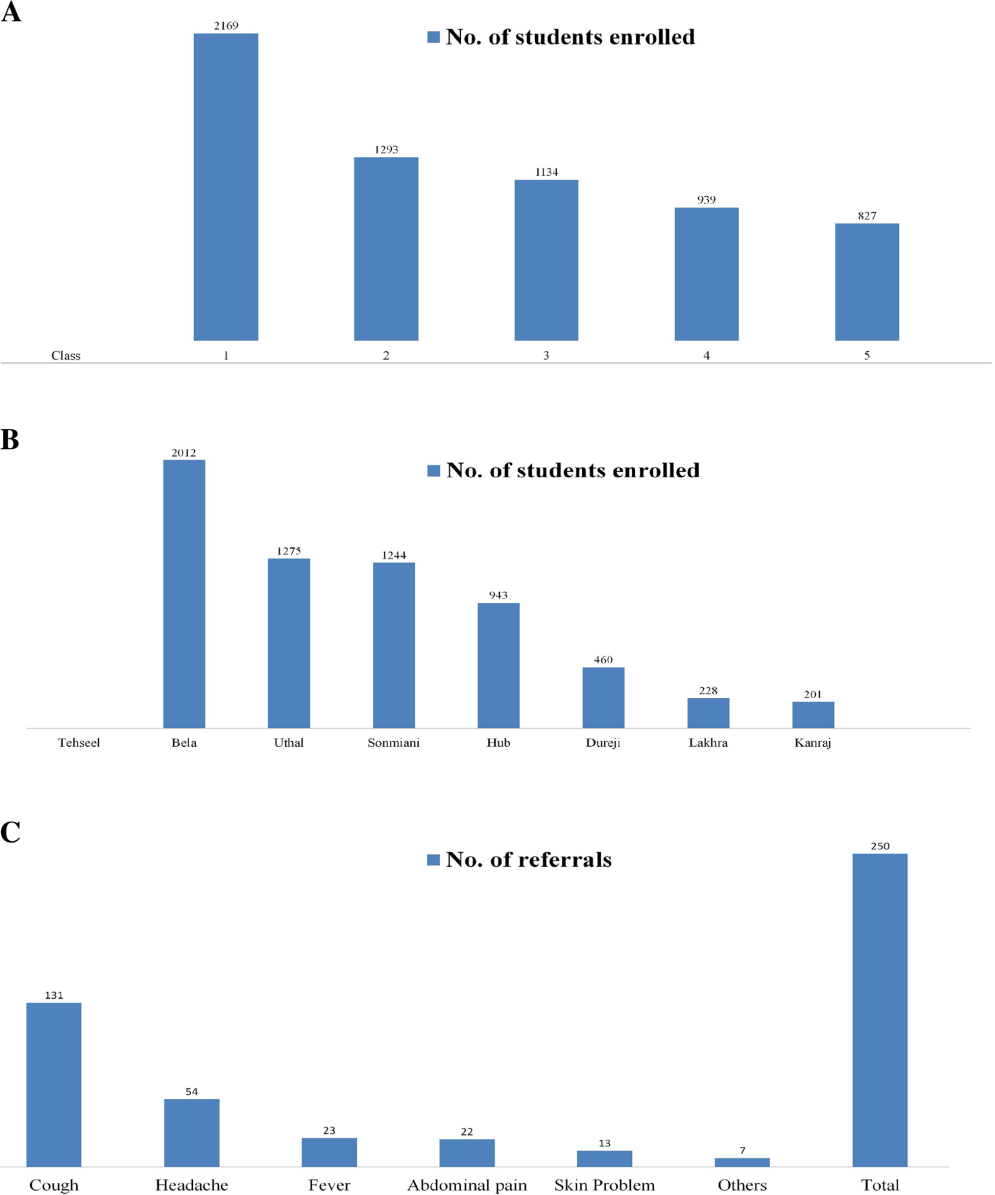
**a** Class-wise distribution of primary school children at Lasbela, District. **b** Tehseel-wise frequency distribution of number of school children. **c** Causes of referral to healthcare facility

Out of 200 Schools visited by field teams, 196 schools were opened, while, 2% of schools (04) remained closed. A total of 6363 students were screened, 56.6% were males and rest 43.4% females. Majority (51.7%) of school children were in between 7–11 years of age. Class wise highest number of school children (34.1%) enrolled in class one, 20.3% in class two, 17.8% in class three, 14.8% in class four and only 13% children in class five. Out of seven tehseels of Lasbela, highest number of children enrolled in primary schools of Bela (31.6%), followed by Uthal (20%), Sonmiani (19.6%) and Hub (14.8%). Around 4% study participants referred to nearest health facility; mainly due to cough, headache and fever. (Table 1 and Fig. 1)

**Table 1:** Age-wise distribution of primary school children at Lasbela, District

Age(yrs)	Frequency	Percent
4–7	1545	24.3
>7–11	3289	51.7
>11–15	1479	10.7
>15	50	.8
Total	6363	100

Based on BMI scores, 44.1% of the study population had normal body mass index (BMI), followed by Mild malnutrition (22.6%), Moderate malnutrition(15.1%), Severe Acute Malnutrition (9.2%) and around 9% overweight/obese, respectively. (Table 2)

**Table 2:** Nutritional Status of primary schoolchildren using BMI scores

Nutritional status	Class					Total (%)
	1	2	3	4	5	
Normal	1035	577	490	373	329	2805 (44.1)
Severe acute malnutrition	181	96	113	112	86	588 (9.2)
Moderate malnutrition	265	198	206	157	133	959 (15.1)
Mild malnutrition	392	324	259	240	221	1436 (22.6)
Overweight	177	63	45	37	46	368 (5.8)
Obese	119	35	21	20	12	207 (3.3)

**Fig. 2:**
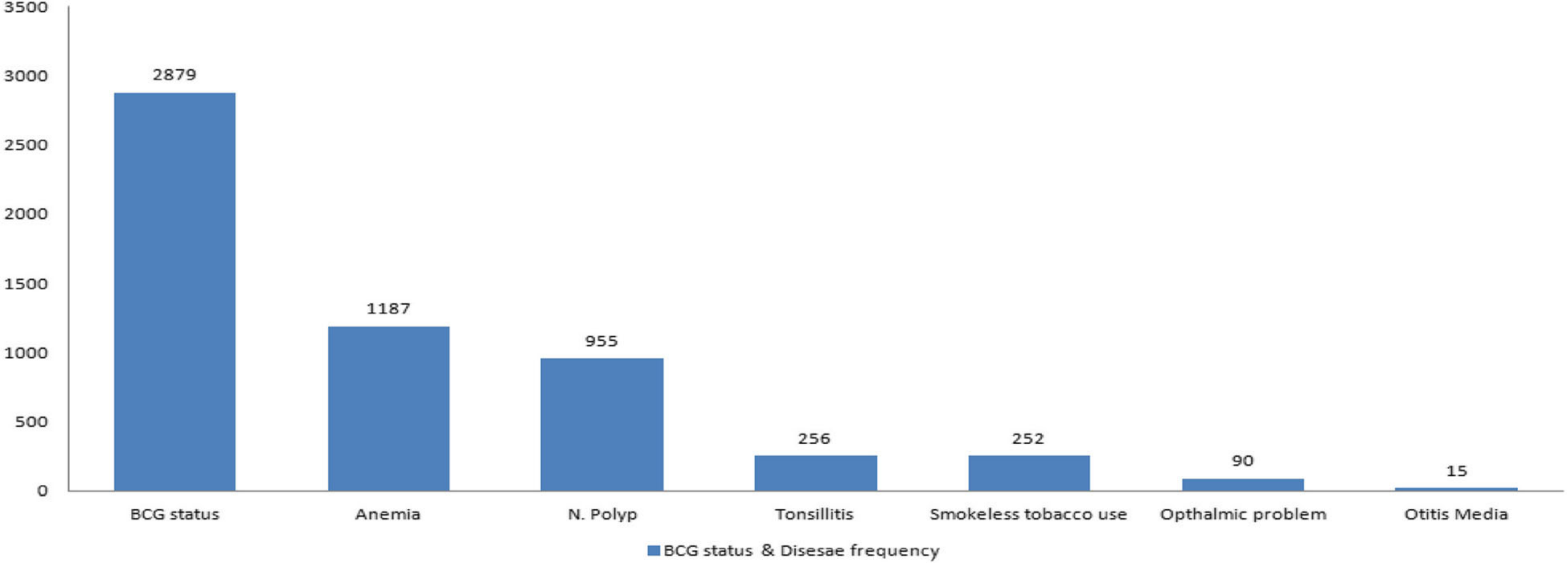
Frequency of clinical manifestations among school going children

More than 19% children had ear nose and throat (ENT) problem followed by Nasal polyp (15%), tonsillitis (4%) and Otitis media (0.2%). Out of 1226 children reported for ENT problems, more than 87% (1076) school going children belonged to Bela tehseel. (Table 3) Clinical anemia was 18.7%; 4% of primary school children informed about eating gutaka / supari; and around 1.4% children had problems related to vision, referred to concerned health facility. Less than 50% of children had scar of BCG vaccination. (Fig. 2)

**Table 3:** Tehseel wise Prevalence of ENT ailments

Tehseel	N. polyp	Tonsillitis	Otitis media	Total
Bela	844	227	5	1076
Hub	30	0	4	34
Sonmiani	68	8	4	80
Kanraj	5	7	0	12
Uthal	3	10	1	14
Lakhra	5	4	1	10
Total	955	256	15	1226

## Discussion

Findings of our study revealed that both ends of malnutrition are concurrent in primary school children at Lasbela. In comparison, estimated prevalence of undernourished children is around five folds higher than obese and overweight category (i.e., 9%). More than 9% children presented with SAM. Only 44% children had normal BMI in our study population. This situation may lead to number of motor and sensory impairments with limited cognitive development among children ultimately could be a huge economic burden if not intervened immediately [6, 8]. Therefore, our major recommendation is to address this issue with multiple intervention plan at public, regulatory and administrative level to reduce harm and inculcate healthy environment in social fabric by sustainable awareness campaigns for parents, teachers, gate keepers, etc, and routine medical checkups for children in collaboration with international organizations who previously had proven track record of similar activities [17–19].

Although, gutka chewing is legally ban in Pakistan; since last two decades the use of smokeless tobacco likewise Gutka/Supari is radically increased in rural communities. Same depicted in our findings with more than 4% primary school children addicted of chewing gutka/supari on daily basis. Considering that majority of children are in between 7-11 years of age and regular use of gutka/supari can even lead to mouth cancers [20]. It is not out of question to suggest proper enforcement of law against use of gutka in general public with special attention on children and their mothers. WHO has introduced MPOWER strategies to implement/incorporate systematic tobacco control program in both urban and rural communities in consultation with concerned regulatory authorities may be feasible in our scenario to control the situation [21].

Immunization coverage in Pakistan varies from 56 to 88% [22]. In current study, only BCG coverage was verified through physical examination of scar on shoulder of children. Around 45% primary school children received BCG. The regulatory authorities need to strengthen their operational approach related to vaccination with contextually more workable plan for optimal coverage.

Alarmingly, high frequency (87.8%) of cases related to ENT problems were recorded in Bela tehseel. It is may be due to mountainous area, unpurified water and low hygiene [23, 24]. Aggressive mass level efforts including medical camps can be beneficial to avoid advance stage ENT related ailments in respective communities.

An incremental trend in school admission is an admirable effort of all stake holders including administrative, regulatory authorities and other partners. Similarly, more efforts are required to escalate the sustainability of these admissions in higher classes with appropriate measures.

Class wise descending trend in terms of enrolment in schools found from class 1 to 5, may be due to massive awareness campaign regarding education in respective territory by regulatory and public authority. On other way, declining number of students in higher class may be due to sociodemographic, low educational performance, and poverty reasons, as most of population of the respective communities was belonging to labor class [25].

## Conclusions

In conclusion, the findings of our study revealed high prevalence of malnutrition, poor oral health including smokeless tobacco use and low BCG coverage among primary school children at Lasbela, Balochistan. Therefore, contextually focused approach to improve awareness, health education and service provision is required to reduce the burden of certain ailments. It is also suggested that future interventions may be planned with integrated approach to identify the causal relationship for further advancement and better incorporation at policy level.

## Abbreviations

BCG: Bacille Calmette Guerin
BMI: Body mass index
ENT: Ear nose throat
LMIC: Low middle income countries
MPOWER: Monitor, protect, offer, warn, enforce, and reduce
NRSP: National Rural Support Program
SAM: Severe acute malnutrition
